# Construction of circ_0071922‐miR‐15a‐5p‐mRNA network in intervertebral disc degeneration by RNA‐sequencing

**DOI:** 10.1002/jsp2.1275

**Published:** 2023-08-24

**Authors:** Yongjin Li, Baobao Wang, Wenzhi Sun, Chao Kong, Junzhe Ding, Feng Hu, Jianhua Li, Xiaolong Chen, Shibao Lu

**Affiliations:** ^1^ Department of Orthopedics Xuanwu Hospital, Capital Medical University Beijing China; ^2^ National Clinical Research Center for Geriatric Diseases Beijing China; ^3^ Spine Center, Department of Orthopaedics, The First Affiliated Hospital of USTC, Division of Life Sciences and Medicine University of Science and Technology of China Hefei Anhui China; ^4^ Department of Orthopedics Tianjin Haihe Hospital Tianjin China

**Keywords:** bioinformatics analysis, circular RNA, ferroptosis, immune response, intervertebral disc degeneration, RNA‐sequencing

## Abstract

**Background:**

Low back pain (LBP) is the main factor of global disease burden. Intervertebral disc degeneration (IVDD) has long been known as the leading reason of LBP. Increasing studies have verified that circular RNAs (circRNAs)‐microRNAs (miRNAs)‐mRNAs network is widely involved in the pathological processes of IVDD. However, no study was made to demonstrate the circRNAs‐mediated ferroptosis, oxidative stress, extracellular matrix metabolism, and immune response in IVDD.

**Methods:**

We collected 3 normal and 3 degenerative nucleus pulposus tissues to conduct RNA‐sequencing to identify the key circRNAs and miRNAs in IVDD. Bioinformatics analysis was then conducted to construct circRNAs‐miRNAs‐mRNAs interaction network associated with ferroptosis, oxidative stress, extracellular matrix metabolism, and immune response. We also performed animal experiments to validate the therapeutic effects of key circRNAs in IVDD.

**Results:**

We found that circ_0015435 was most obviously upregulated and circ_0071922 was most obviously downregulated in IVDD using RNA‐sequencing. Then we observed that hsa‐miR‐15a‐5p was the key downstream of circ_0071922, and hsa‐miR‐15a‐5p was the top upregulated miRNA in IVDD. Bioinformatics analysis was conducted to predict that 56 immunity‐related genes, 29 ferroptosis‐related genes, 23 oxidative stress‐related genes and 8 ECM‐related genes are the targets mRNAs of hsa‐miR‐15a‐5p. Then we constructed a ceRNA network encompassing 24 circRNAs, 6 miRNAs, and 101 mRNAs. Additionally, we demonstrated that overexpression of circ_0071922 can alleviate IVDD progression in a rat model.

**Conclusions:**

The findings of this study suggested that circ_0071922‐miR‐15a‐5p‐mRNA signaling network might affect IVDD by modulating the nucleus pulposus cells ferroptosis, oxidative stress, ECM metabolism, and immune response, which is an effective therapeutic targets of IVDD.

## Graphical abstract

We confirmed that circ_0015435 was most obviously upregulated in IVDD using RNA‐sequencing and RT‐PCR experiment. Then we constructed circ_0071922‐miR‐15a‐5p‐mRNA signaling network encompassing 24 circRNAs, 6 miRNAs, and 101 mRNAs and found its might affect IVDD by modulating the nucleus pulposus cells ferroptosis, oxidative stress, ECM metabolism, and immune response by bioinformatics analysis. Additionally, we also demonstrated that overexpression of circ_0071922 can alleviate IVDD progression in a rat model.

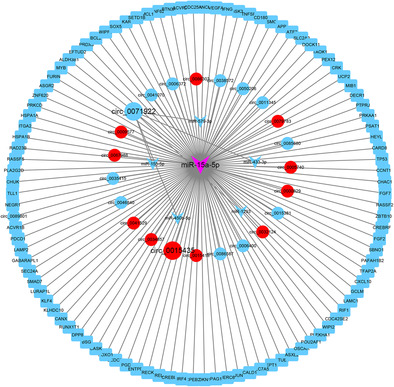

## 1 INTRODUCTION

Low back pain (LBP) is a global public hygienic issue that affects up to 80% of individuals, imposing a great impact on the life and work of the young and middle‐aged labor force.^1^, ^2^, ^3^ From 1996 to 2016, US spending on health care increased significantly, of which LBP spending accounted for the highest proportion among 154 diseases, which brought a serious physical and mental health problems and huge economic losses to human beings.^4^ A series of literatures reported by the Lancet calls for global action on LBP.^5^, ^6^, ^7^ Extensive evidence have revealed that intervertebral disc degeneration (IVDD) is the most common chronic skeletal muscle degeneration disease and a primary reason of triggering LBP or secondary neurological deficits.^8^, ^9^, ^10^ To our knowledge, IVDD is a pathological condition characterized by difficult early diagnosis, limited treatment strategies and poor prognosis.^11^, ^12^ Present treatments for IVDD diseases range from drugs to invasive surgical operations treatments, but these treatments have limited efficacy and can neither block the pathological progress of IVDD nor restore the own function of the intervertebral disc.^12^, ^13^, ^14^ For this phenomenon, there is a compelling need to develop a therapeutic approach with improved efficacy. Therefore, a better identifying, interpreting and understanding of the pathomechanism of IVDD might develop more targeted treatments.

Recently, molecular treatments strategies such as targeted gene and RNA treatments have shown promising therapeutic potential for alleviating or reversing the degenerative process of IVDD.^14^, ^15^, ^16^ To study this issue, we first analyze the pathological process and pathogenesis of IVDD. Intervertebral disc is the most important functional unit of the spine, and the nucleus pulposus is an important part of the intervertebral disc, which can carry complex mechanical loads, allowing mild movement of the spine in multiple directions, including axial flexion, compression, and rotation.^12^, ^17^, ^18^ Based on this anatomical basis, the degeneration of nucleus pulposus is often the earliest stage of IVDD.^8^ Many scholars reported that the homeostasis disbalance of extracellular matrix (ECM), nucleus pulposus cells death (such as cuproptosis and ferroptosis), oxidative stress, and immune response in nucleus pulposus were the key pathomechanism of IVDD.^8^, ^12^, ^14^, ^19^, ^20^ These pathological processes interact with each other and jointly play an important role in the occurrence and progression of IVDD.^8^, ^14^ Taken together, studying the gene and RNA‐mediated the pathological processes of IVDD is extremely important for improving LBP and the quality of life of patients.

Non‐coding RNA (ncRNA) family includes circular RNAs (circRNAs), long‐chain ncRNAs (lncRNAs) and microRNAs (miRNAs), most of which cannot encode proteins and can regulate cell function and homeostasis.^21^ Our previous studies have unveiled that the differential expression of circRNA, miRNA and mRNA is a common phenomenon during IVDD, and they often regulate the progression of IVDD via constituting circRNA‐miRNA‐mRNA network.^22^, ^23^, ^24^, ^25^, ^26^ On the mechanism, the miRNA‐guided RNA‐induced silencing complex (RISC) targets mRNA, and miRNA accelerates mRNA degradation or prevents its translation by directly binding to the 3′ untranslated region (3′UTR) of target gene mRNA.^27^ CircRNAs served as competitive endogenous RNAs (ceRNAs), compete for miRNA targeting and binding to RISC complexes, and isolate the miRNA‐RISC complexes from target genes, thereby positively regulating the expression of target genes mRNA.^27^, ^28^ Growing proof have shown that targeted gene and circRNA treatments can significantly alleviate IVDD via mediating ECM homeostasis, ferroptosis, and oxidative stress, etc.^24^, ^25^, ^26^, ^29^ Thus, it is critical to explore novel IVDD‐related circRNA, miRNA and mRNA that can mediate the pathological process of IVDD.

Currently, we intend to identify the expression profiles of circRNAs and miRNAs in IVDD by conducting high throughput RNA‐sequencing. Then, the circRNA‐miRNA‐mRNA network was constructed by integrated bioinformatics analysis, which provides a potential strategy for IVDD diseases treatment.

## 2 MATERIALS AND METHODS

### 2.1 Human samples collection and grouping

The protocol of this study was supervised and approved by the Ethics Committee of the Xuanwu Hospital of Capital Medical University and First Affiliated Hospital of USTC. Three normal NP tissues and three degenerative NP tissues were included in this study for RNA‐sequencing. Additionally, we once again collected seven normal NP tissues and ten degenerative NP tissues for quantitative real‐time PCR (qRT‐PCR) verification. The basic information and characteristics for all patients were listed in Table 1. All patients signed the informed consent form before the samples collection. We confirm that all methods were carried out in accordance with relevant guidelines and regulations such as Declaration of Helsinki. The degree of nucleus pulposus degeneration was determined according to Pfirrmann's grade.^30^ The normal tissues with Pfirrmann grade I–II were collected from patients suffering from fresh thoracolumbar fracture or scoliosis, whereas the degenerative NP tissues with Pfirrmann grade III‐V were collected from IVDD patients with lumbar disc herniation or lumbar spinal stenosis or lumbar spondylolisthesis.

**TABLE 1:** The basic information and characteristics for patients.

Samples	Type	Gender	Age (years)	Diagnosis	Tissues	Pfirrmann grade
Sample 1	Normal 1	Male	18	Thoracolumbar fracture	NP	I
Sample 2	Normal 2	Female	20	Thoracolumbar fracture	NP	II
Sample 3	Normal 3	Male	24	Thoracolumbar fracture	NP	II
Sample 4	IVDD 1	Female	55	Lumbar disc herniation	NP	IV
Sample 5	IVDD 2	Female	74	Lumbar disc herniation	NP	V
Sample 6	IVDD 3	Male	68	Lumbar disc herniation	NP	V
Sample 7	Normal 1	Male	30	Thoracolumbar fracture	NP	II
Sample 8	Normal 2	Male	26	Thoracolumbar fracture	NP	II
Sample 9	Normal 3	Male	15	Thoracolumbar fracture	NP	I
Sample 10	Normal 4	Female	20	Scoliosis	NP	I
Sample 11	Normal 5	Male	18	Scoliosis	NP	II
Sample 12	Normal 6	Female	24	Scoliosis	NP	II
Sample 13	Normal 7	Female	16	Scoliosis	NP	I
Sample 14	IVDD 1	Female	78	Lumbar spinal stenosis	NP	V
Sample 15	IVDD 2	Female	82	Lumbar spinal stenosis	NP	V
Sample 16	IVDD 3	Female	70	Lumbar spinal stenosis	NP	V
Sample 17	IVDD 4	Male	68	Lumbar spinal stenosis	NP	IV
Sample 18	IVDD 5	Female	64	Lumbar disc herniation	NP	IV
Sample 19	IVDD 6	Female	43	Lumbar disc herniation	NP	III
Sample 20	IVDD 7	Male	56	Lumbar disc herniation	NP	IV
Sample 21	IVDD 8	Female	45	Lumbar spondylolisthesis	NP	III
Sample 22	IVDD 9	Male	60	Lumbar spondylolisthesis	NP	IV
Sample 23	IVDD 10	Female	69	Lumbar spondylolisthesis	NP	IV

### 2.2 Analysis of the circRNAs and miRNAs profile

All the NP tissues were immediately frozen in liquid nitrogen. The total RNA was extracted from three normal and three degenerative NP tissues with TRIzol reagent (Invitrogen, Carlsbad, CA, USA). The genomic DNA and ribosomal RNA were removed from total RNA using RNase‐free DNase I and Ribo‐off rRNA depletion kit (Suzhou, Genewiz, China), respectively. We then quantified the RNAs in each tissue using a NanoDrop ND‐2000 spectrophotometer. To improve circRNAs enrichment, we added Rnase R (Epicentre, Madison, WI, USA) to remove linear RNAs. Subsequently, RNA sequencing were performed by Suzhou Genewiz Biotechnology Company (Suzhou, China). The acquired array images were analyzed via Bcl2fastq (v2.17.1.14) to conduct Base Calling for initial quality analysis and obtain the raw sequencing data. FastQC software (V0.11.4) was used to evaluate the quality of sequencing data. The adapter sequences and low quality data were filtered to generate clean data using Cutadapt (version 1.9.1). BWA (Version 0.7.12) was used to compare clean data to the reference genome sequence to generate sequence alignment files and visualized by Integrative Genomics Viewer. CIRI (V2.06) software was used to predict the position information before and after circRNA formation to identify circRNAs. As for the raw miRNA sequencing data, we compared the sequences of clean data with known miRNAs in the miRBase database and annotated known miRNAs. We then compared the sequenced sequences with the whole genome sequences of human, and predicted new miRNAs through folding models. For all circRNAs and miRNAs from normal and degenerative NP tissues, |log_2_ fold‐change (FC)| > 1 and *p* values < 0.05 were considered to be significantly different.

### 2.3 Collection of oxidative stress‐related genes (OSRGs), ferroptosis‐related genes (FRGs), cuproptosis‐related genes (CRGs), immunity‐related genes (IRGs), and ECM related genes

The lists of ECM‐related genes were obtained from the Reactome database (Version 79, https://reactome.org/) and HUGO gene.^31^ The lists of FRGs were obtained from the FerrDb V2 database (http://www.zhounan.org/ferrdb/).^32^ The lists of CRGs were obtained from our previous study.^33^, ^34^ The lists of OSRGs and IRGs were obtained from the molecular signatures database (https://www.gsea-msigdb.org/gsea/msigdb/index.jsp)^35^ and immunology database and analysis portal (immport, https://www.immport.org/home).^36^ All the genes were listed in Table S1.

### 2.4 Exploring the interaction between the circRNAs and miRNAs

CircBank is a comprehensive database of human circRNAs, which can also be used to analyze the interaction between the circRNAs and their downstream miRNAs (http://www.circbank.cn/).^37^ We used CircBank database to predict the downstream miRNAs of the key circRNAs as well as the upstream circRNAs of the key miRNAs. Subsequently, we predicted the overlapping key miRNAs via merging CircBank and RNA‐seq. StarBase database (http://starbase.sysu.edu.cn/) including more than 9000 miRNA‐circRNA regulatory relationships.^38^ The upstream overlapping circRNAs of the key miRNA were predicted via merging starBase, CircBank databases and RNA‐seq.

### 2.5 Prediction the downstream mRNAs of the key miRNA

miRTarBase (https://miRTarBase.cuhk.edu.cn/)^39^ database is an informative resource for experimentally validated miRNA‐target interactions, so we used this database to predict the potential target genes of the key miRNA. Then we merged miRTarBase, FRGs, OSRGs, IRGs and CRGs to select the key mRNAs.

### 2.6 Construction of circRNA‐miRNA‐mRNA ceRNA regulatory network

To further clearly elucidate the interactions between circRNAs and mRNAs, a circRNA‐miRNA‐mRNA ceRNA regulatory network was constructed by combining data from circRNAs and mRNAs with miRNA data. The above screened circRNAs, miRNAs and mRNAs were used to construct circRNAs‐miRNAs‐mRNAs network using Cytoscape software version 3.7.1 (https://cytoscape.org/).^40^

### 2.7 Construction of Protein–Protein Interaction (PPI) network

The correlation networks of multiple functional proteins were analyzed using the Search Tool for the Retrieval of Interacting Genes (STRING) database (https://cn.string-db.org/).^41^ Then we input the host genes of circRNAs into the multiple protein section of STRING database to construct the PPI network and further visualized using the cytoHubba plug‐in of Cytoscape software.^40^ Furthermore, the MCC method was used to assess the PPI score.

### 2.8 Functional and pathway enrichment analysis of the host genes of circRNAs

To predict the potential biological functions of the host genes of circRNAs, we conducted Gene Ontology (GO; www.geneontology.org) functional and Kyoto Encyclopedia of Genes and Genomes (KEGG; https://www.genome.jp/kegg/) pathway enrichment analysis. GO is a bioinformatics analysis tool, including three aspects: biological process (BP), cellular component (CC), and molecular function (MF). KEGG is a comprehensive database that integrates genomic, chemical and systemic functional information, which is used to predict the key signaling pathways.^42^ The *p* value represents the significance of the enriched GO and KEGG terms among the host genes of circRNAs. The results of Go and KEGG analysis were output by the online bioinformatics analysis tool (http://www.bioinformatics.com.cn).

### 2.9 qRT‐PCR

Total RNAs were extracted from multiple normal and degenerative NP tissues using TRIzol reagent (Life Technologies, Thermo Fisher Scientific, USA). Micro spectrophotometer (Nano‐300, Allsheng, Hangzhou, China) was used to detect the concentration and purity of RNA. Then the RNA were reverse transcribed into cDNA using the Transcriptor First Strand cDNA Synthesis Kit (Takara Biotechnology, Otsu, Japan) following the manufacturer's protocol. Next, the PCR reactions were performed on ABI 7500 system (Applied Biosystems, USA) using SYBR Green Kit Master Mix and primers. The relative expression levels of circ_0071922 was measure during the 2^−△△Ct^ method.

### 2.10 Construction and treatment of rats IVDD model

A total of 18 Sprague–Dawley rats with 3 months were obtained from the American Charles River Laboratories to perform animal experiments. The animal's experiments were supervised and approved by the Ethics Committee of the Xuanwu Hospital of Capital Medical University. We confirm that all methods were carried out in accordance with ARRIVE guidelines. They were randomly divided into three groups, of which 12 rats underwent needle puncture and other 6 rats without any intervention as control. The rats were anesthetized through intraperitoneal injection of 90 mg/kg ketamine and 10 mg/kg xylazine. Under the guidance of fluoroscopy, 31G needle was used to puncture the Co7/8 and Co9/10 intervertebral discs of the rat tail from the dorsal side, and passed through the annulus fibrosus, and followed by inserting into the nucleus pulposus region about 1.5 mm, and then rotate 180° in the axial direction and held for 10s. The Co8/9 was left undisturbed as a control. One day and 4 weeks after the puncture, 2 μL experimental or control virus vector solutions were slowly injected into the intervened intervertebral discs.

### 2.11 X‐ray examination, histological assessment, and immunohistochemical staining

X‐ray examination was conducted at 4 and 8 weeks after the needle puncture. Based on our previous method,^26^ the rats were killed by intraperitoneal injection of over‐dose pentobarbital sodium, and the NP tissues were collected. Then the NP tissues were fixed in 4% paraformaldehyde for 48 h, decalcified in ethylene diamine tetraacetic acid (EDTA), and embedded in paraffin. Subsequently, the NP tissues were cut into a thickness of 5 μm sections along the midsagittal plane for HE and SO staining. The histological scoring results were quantitatively evaluated according to our previous method.^26^ With regard to immunohistochemical staining, the dewaxed NP tissues sections were incubated in EDTA to retrieve the antigen. After blocking with 1% goat serum albumin, the sections were incubated with a primary antibody against BCL2 (ab182858, Abcam) for 2 h at 4°C overnight. The sections were then incubated with secondary antibody for 1 h at room temperature. The figures were obtained by the fluorescence microscope (Leica).

### 2.12 Statistical analysis

We carried out at least three independent experiments. The data was analyzed using GraphPad Prism software 6 version. The statistical significance between the two groups was compared by an unpaired Student's *t‐*test, whereas the differences between more than two groups were assessed by one‐way analysis of variance followed by Turkey's multiple comparisons test. Results are presented as mean ± standard deviation. *p*‐value <0.05 was considered to be statistically significant.

## 3 RESULTS

### 3.1 Selecting the DECs in IVDD patients by RNA‐sequencing

**FIGURE 1:**
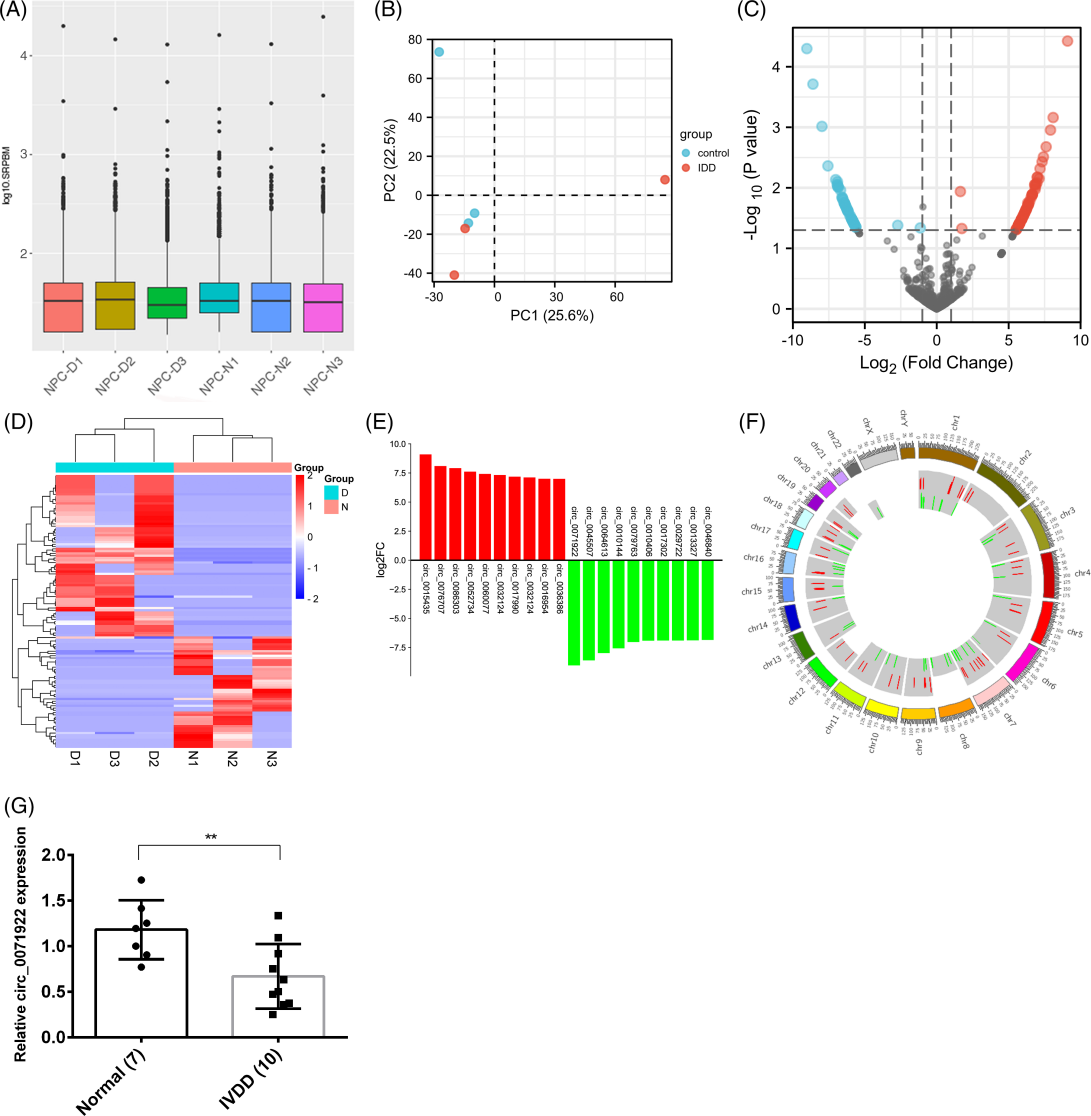
Selecting the DECs in IVDD patients by RNA‐sequencing. (A) Box plot. The abscissa is the sample name, the ordinate is log10 (SRPBM), and the box plot for each region is against five statistics (top to bottom are maximum, upper quartile, median, lower quartile and the minimum value, respectively). (B) PCA plot. The horizontal and vertical coordinates reflect the relative distance among different samples. (C) Volcano plot of the DECs. (D) Cluster heatmap of the DECs. (E) Bar plot. The *X*‐axis indicates the DECs and the *Y*‐axis indicates log2 fold change. Red means upregulation, above the *X*‐axis; green means downregulation, below the *X*‐axis. (F) Circos plot. The distribution of DECs on chromosomes. (G) The expression level of circ_0071922 in IVDD was detected using qRT‐PCR.

Bulk RNA sequencing (RNA‐seq) techniques and bioinformatics tools have enhanced our understanding of circRNA, which is an invaluable tool for identifying DECs between normal and degenerative nucleus pulposus tissues.^43^, ^44^ Considering that it is difficult to distinguish the boundary between the nucleus pulposus and annulus fibrosus, we collected gelatinous tissue from the intervertebral disc central region as the nucleus pulposus.^45^ To explore the expression characteristics of circRNAs in IVDD patients, we conducted RNA‐seq. The box plot unveiled that the median of the overall data of the 6 samples is on the same level after data standardization, indicating that the standardized result is very good (Figure 1A). The results of Principal component analysis (PCA) displayed that the within‐group differences was smaller, but the differences between control and IVDD groups was obvious, suggesting that the samples selection is is correct and reasonable (Figure 1B). A total of 6886 circRNAs were discovered in IVDD patients using RNA‐seq. The cut‐off value of the DECs screening was |log_2_ (FC)| > 1 and *p* values <0.05. We identified 264 upregulated and 168 downregulated DECs, which was shown in volcano plot (Figure 1C) and heatmap (Figure 1D). Among them, 129 circRNAs have been annotated in the circbase database (http://www.circbase.org/), the other 303 circRNAs were called NovelcircRNAs (have not been annotated). The detail information of 129 DECs were listed in Tables S1 and S3. Bar plot further visualize the differential expression of top 10 upregulated and downregulated circRNAs, of which circ_0015435 was the top upregulated and circ_0071922 was the top downregulated DECs in IVDD, respectively (Figure 1E). Moreover, we also demonstrated that the expression level of circ_0071922 was significantly downregulated in IVDD using qRT‐PCR (Figure 1G). Additionally, the distribution of DECs on chromosomes was shown in circos plot (Figure 1F).

### 3.2 PPI analysis of the host genes of circRNAs

**FIGURE 2:**
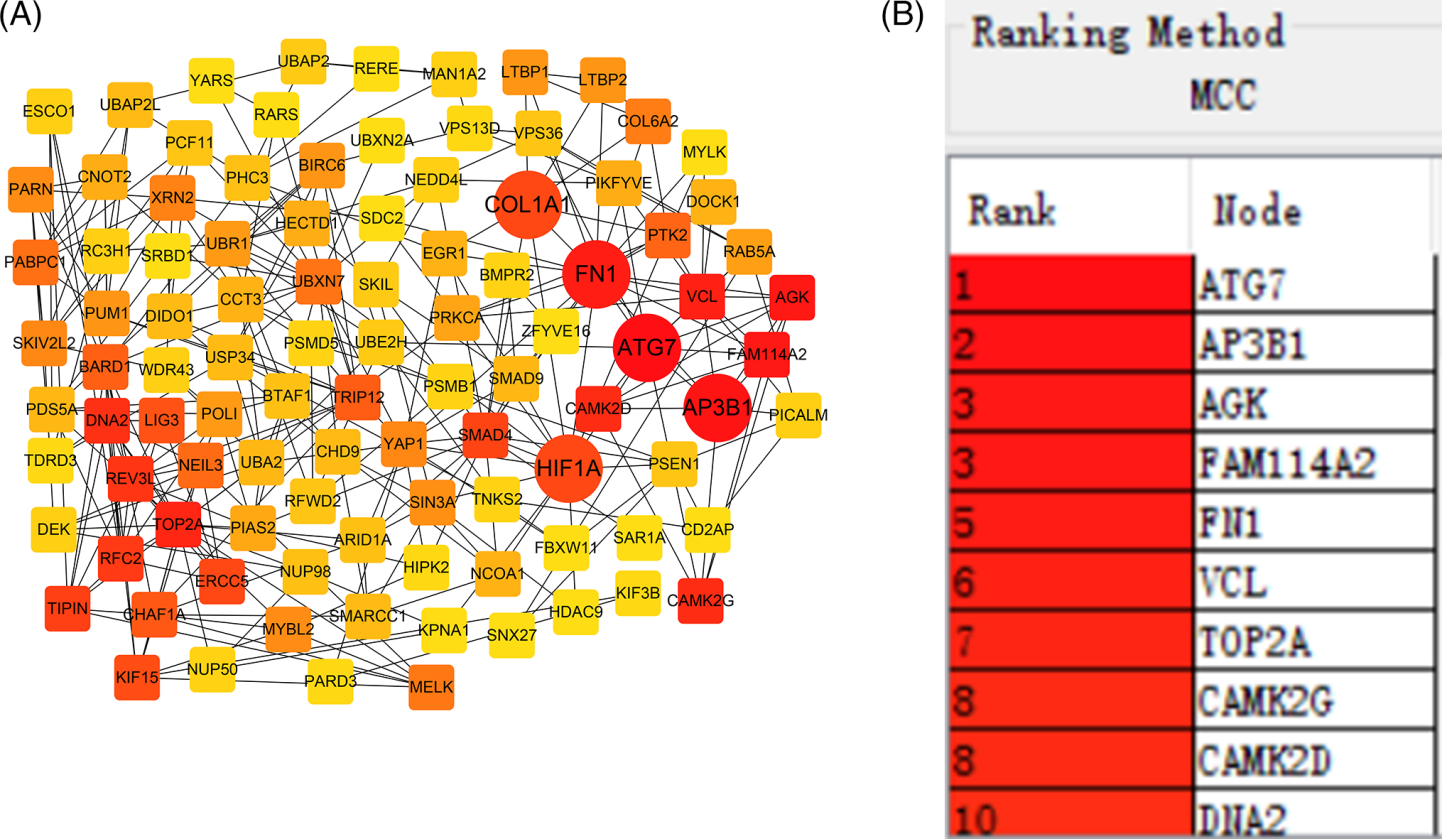
PPI analysis of the host genes of circRNAs. (A) Cytoscape software was used to visualize the host genes of circRNAs. (B) The top 10 ranked genes from PPI network.

A total of 432 host genes of circRNAs were found in this study, which were listed in Table S3. Subsequently, we mapped the 432 genes into STRING website to explore the interaction among them. The top 100 genes were extracted to construct PPI network by Cytoscape software. Among them, the first and second ranked were autophagy related gene 7 (ATG7) and adaptor protein complex 3 beta 1 (AP3B1) (Figure 2A,B), so they were identified as key hub genes. Other top‐ranked genes include fibronectin 1 (FN1), hypoxia inducible factor 1 subunit alpha (HIF1A), and collagen type 1 alpha 1(COL1A1) (Figure 2A,B).

### 3.3 Identifying the functions of the host genes of circRNAs

**FIGURE 3:**
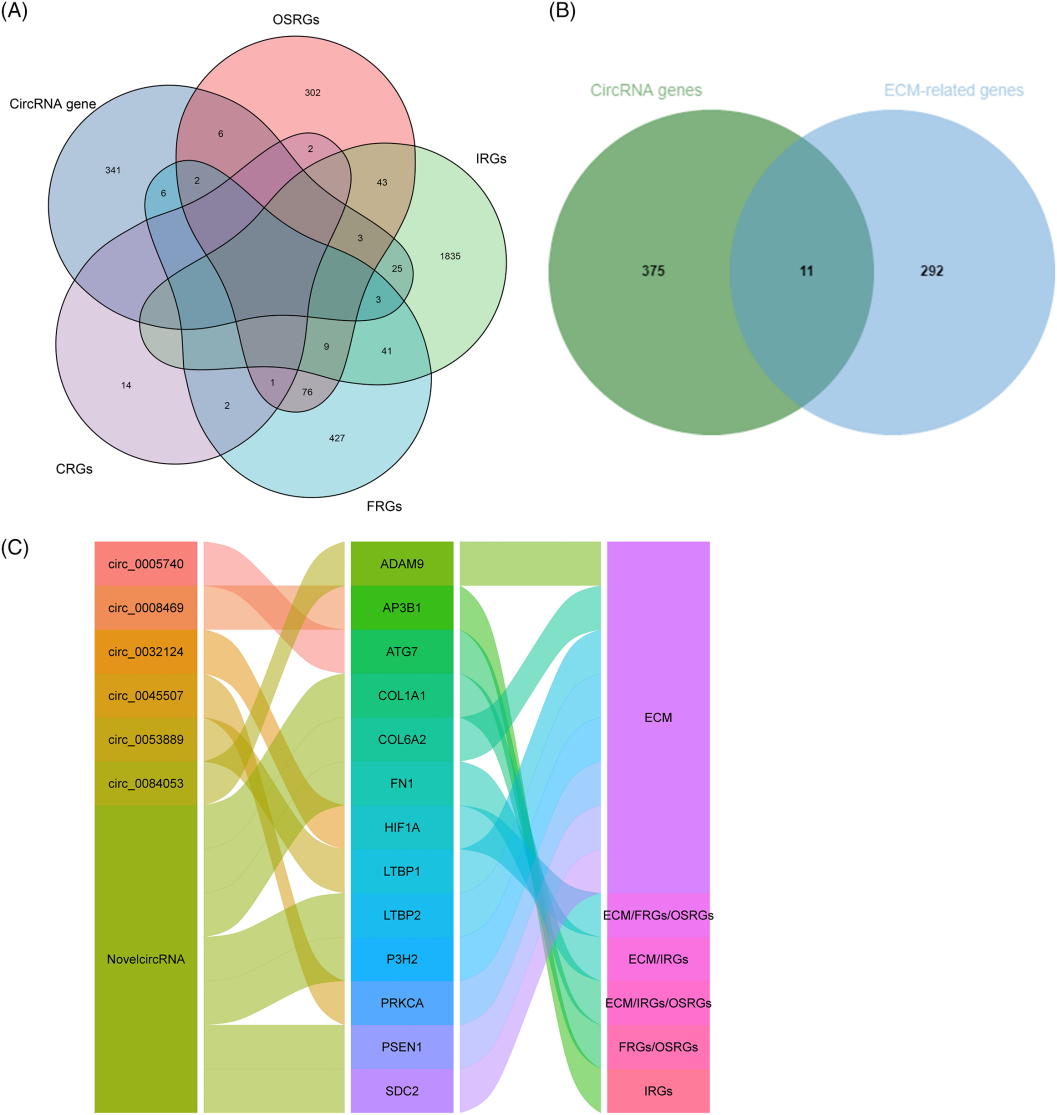
Identifying the functions of the host genes of circRNAs. (A) The host genes of circRNAs related to IRGs, FRGs, CRGs, and OSRGs ECM‐related genes were selected via Venn diagram. (B) The host genes of circRNAs related to ECM‐related genes were selected via Venn diagram. (C) The alluvial plot was used to visualize the key host genes, their corresponding circRNAs, and the functions of the host genes of circRNAs.

To identify the key host genes of circRNAs and their functions, we obtained IRGs, FRGs, CRGs, OSRGs, and ECM‐related genes from different databases. Among the host genes, we found 11 ECM‐related genes, including PRKCA, COL1A1, PSEN1, COL6A2, ADAM9, P3H2, LTBP2, FN1, LTBP1, HIF1A, and SDC2 (Figure 3B,C). Interestingly, ATG7 and HIF1A were also a FRGs and OSRGs, FN1 and AP3B1 were also a IRGs, and COL1A1 was also a IRGs and OSRGs (Figure 3A–C). However, no CRGs was found in the host genes. Furthermore, the alluvial plot was used to visualize the above key host genes, their corresponding circRNAs, and the functions of the host genes of circRNAs (Figure 3C). Taken together, the host genes of circRNAs might link to ferroptosis, oxidative stress, autophagy, ECM metabolism, and immune response, etc.

### 3.4 Functional and pathway enrichment analysis of the host genes of circRNAs

**FIGURE 4:**
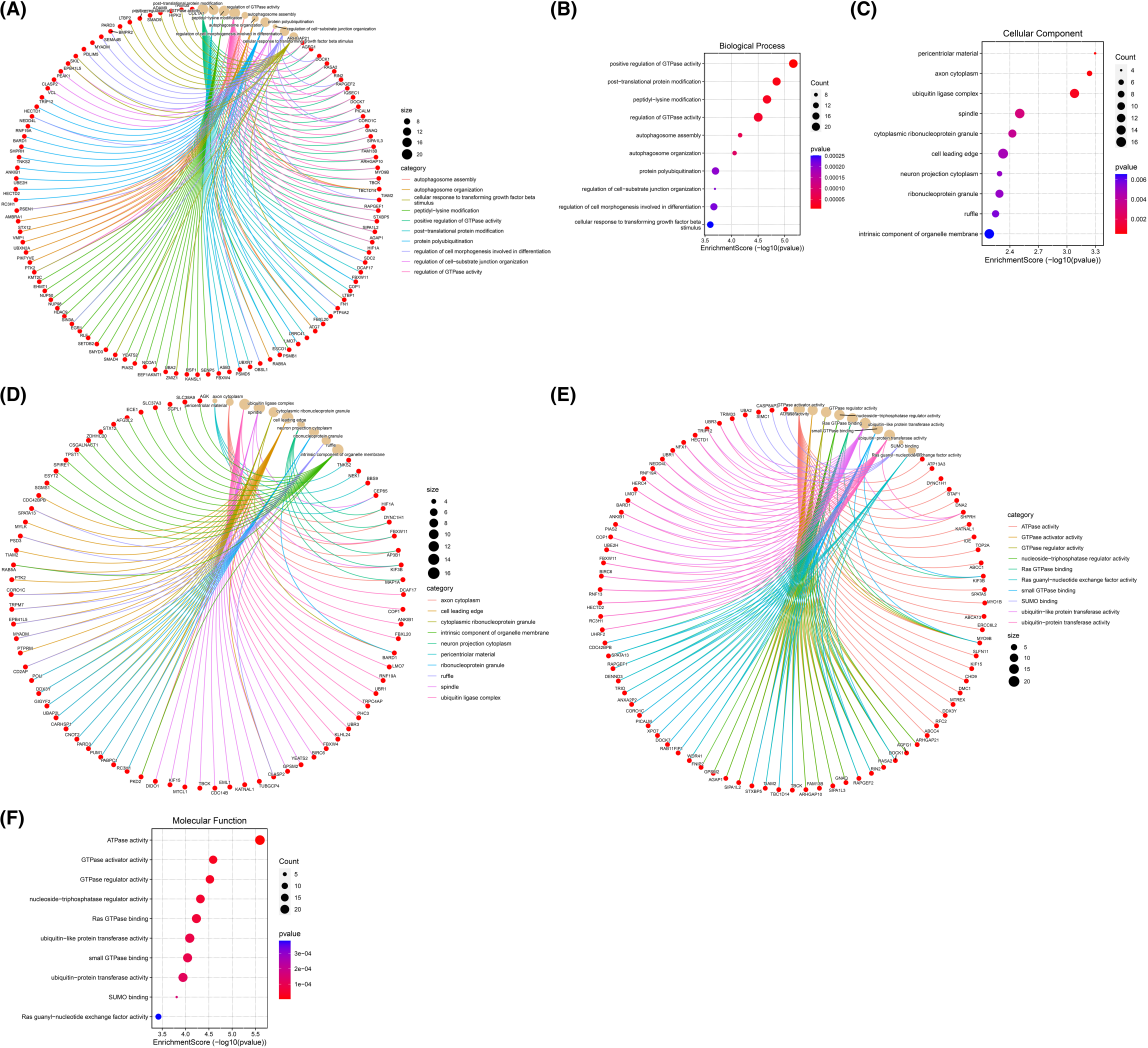
Go functional enrichment analysis of the host genes of circRNAs. Go consists of biological process (BP), cell components (CC), and molecular function (MF). (A) BP cnetplot. (B) BP enrichment score dotplot. (C) CC cnetplot. (D) CC enrichment score dotplot. (E) MF cnetplot. (F) MF enrichment score dotplot.

GO functional and KEGG pathway enrichment analysis is crucial for elucidating the potential roles of high‐throughput omics data. To further predict their functions, we subsequently performed GO and KEGG analysis for the 432 host genes of circRNAs. There were as many as 200 terms enriched in the BP, of which the following top 10 terms are relevant to this study: “positive regulation of GTPase activity; post‐translational protein modification; peptidyl‐lysine modification; regulation of GTPase activity; autophagosome assembly; autophagosome organization; protein polyubiquitination; regulation of cell‐substrate junction organization; regulation of cell morphogenesis involved in differentiation; cellular response to transforming growth factor beta stimulus” (Figure 4A,B). The CC were mainly enriched in “pericentriolar material; axon cytoplasm; ubiquitin ligase complex; spindle; cytoplasmic ribonucleoprotein granule; cell leading edge; neuron projection cytoplasm; ribonucleoprotein granule; ruffle; intrinsic component of organelle membrane” (Figure 4C,D). The predominant top 10 MF terms were as follows: ATPase activity; GTPase activator activity; GTPase regulator activity; nucleoside‐triphosphatase regulator activity; Ras GTPase binding; ubiquitin‐like protein transferase activity; small GTPase binding; ubiquitin‐protein transferase activity; SUMO binding; Ras guanyl‐nucleotide exchange factor activity (Figure 4E,F).

**FIGURE 5:**
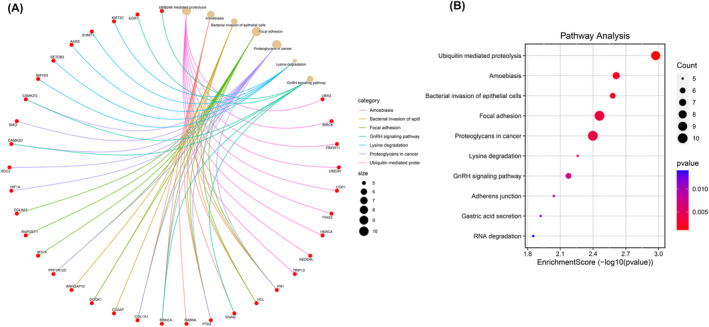
KEGG pathway enrichment analysis of the host genes of circRNAs. (A) KEGG cnetplot. (B) KEGG enrichment score dotplot.

As for KEGG analysis, these host genes were involved in 21 KEGG pathways, the top 10 terms were “Ubiquitin mediated proteolysis; Amoebiasis; Bacterial invasion of epithelial cells; Focal adhesion; Proteoglycans in cancer; Lysine degradation; GnRH signaling pathway; Adherens junction; Gastric acid secretion; RNA degradation” (Figure 5A,B). These results unveiled that many host genes of DECs might participate in the occurrence and progression of IVDD by regulating different pathological process.

### 3.5 Identification of the downstream miRNAs of circ_0015435 and circ_0071922 related to IVDD

**FIGURE 6:**
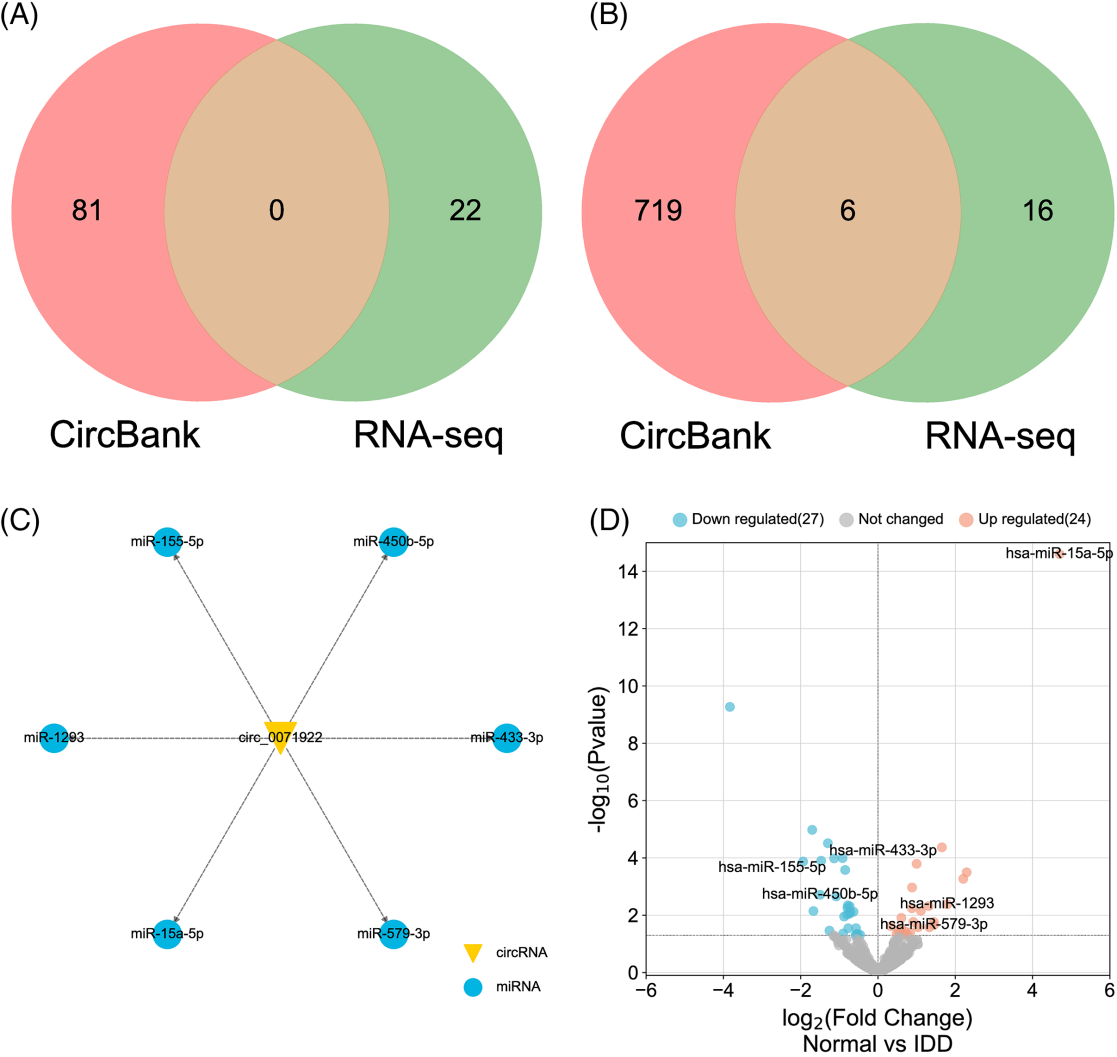
Identification of the downstream miRNAs of circ_0015435 and circ_0071922 related to IVDD. (A) Identification of the downstream miRNAs of circ_0015435 related to IVDD. (B) Identification of the downstream miRNAs of circ_0071922 related to IVDD. (C) CircRNA target miRNAs network. The circRNA is in the inner circle and the miRNAs is in the outer circle. (D) Volcano plot. Green points indicate downregulated miRNAs (left side), gray points indicate no change of miRNAs expression (middle), and red points indicate upregulated miRNAs (right side).

As the powerful genes regulators, increasing studies have uncovered that circRNAs primarily regulate the miRNA‐mRNA pathway via directly binding to miRNA response elements.^24^, ^25^, ^26^, ^29^ Therefore, it is very important to clarify the interaction between circRNAs and miRNAs. Circbank is publicly available database included more than 140 000 human annotated circRNAs, which can be used to analyze the interaction between the circRNAs and their downstream miRNAs.^38^ Given that circ_0015435 was the top upregulated and circ_0071922 was the top downregulated DECs in IVDD, we then used CircBank database to predict the downstream miRNAs of circ_0015435 and circ_0071922. To further select the downstream miRNAs of circ_0015435 and circ_0071922 related to IVDD, we combined the results predicted by the CircBank database with the results obtained by RNA‐seq. However, no overlapped downstream miRNAs of circ_0015435 was found between RNA‐seq and CircBank (Figure 6A), suggesting that circ_0015435 might regulate IVDD in a miRNA‐independent manner. A total of 6 overlapped downstream miRNAs of circ_0071922 related to IVDD were predicted through Venn diagram (Figure 6B). Based on the corresponding regulatory relationship, a circ_0071922‐miRNAs network was constructed as shown in Figure 6C. Moreover, volcano plot was used to visualize the differential expression of these 6 miRNAs, of which hsa‐miR‐15a‐5p, hsa‐miR‐1293, and hsa‐miR‐579‐3p were significantly upregulated, while hsa‐miR‐433‐3p, hsa‐miR‐155‐5p, and hsa‐miR‐450b‐5p were significantly downregulated in IVDD (Figure 6D). Among them, hsa‐miR‐15a‐5p was the top upregulated miRNAs in IVDD (Figure 6D). Taken together, we speculated that circ_0071922 was the key circRNA, and hsa‐miR‐15a‐5p was the key miRNA in IVDD.

### 3.6 Identification of the upstream circRNAs and downstream mRNAs of miR‐15a‐5p

**FIGURE 7:**
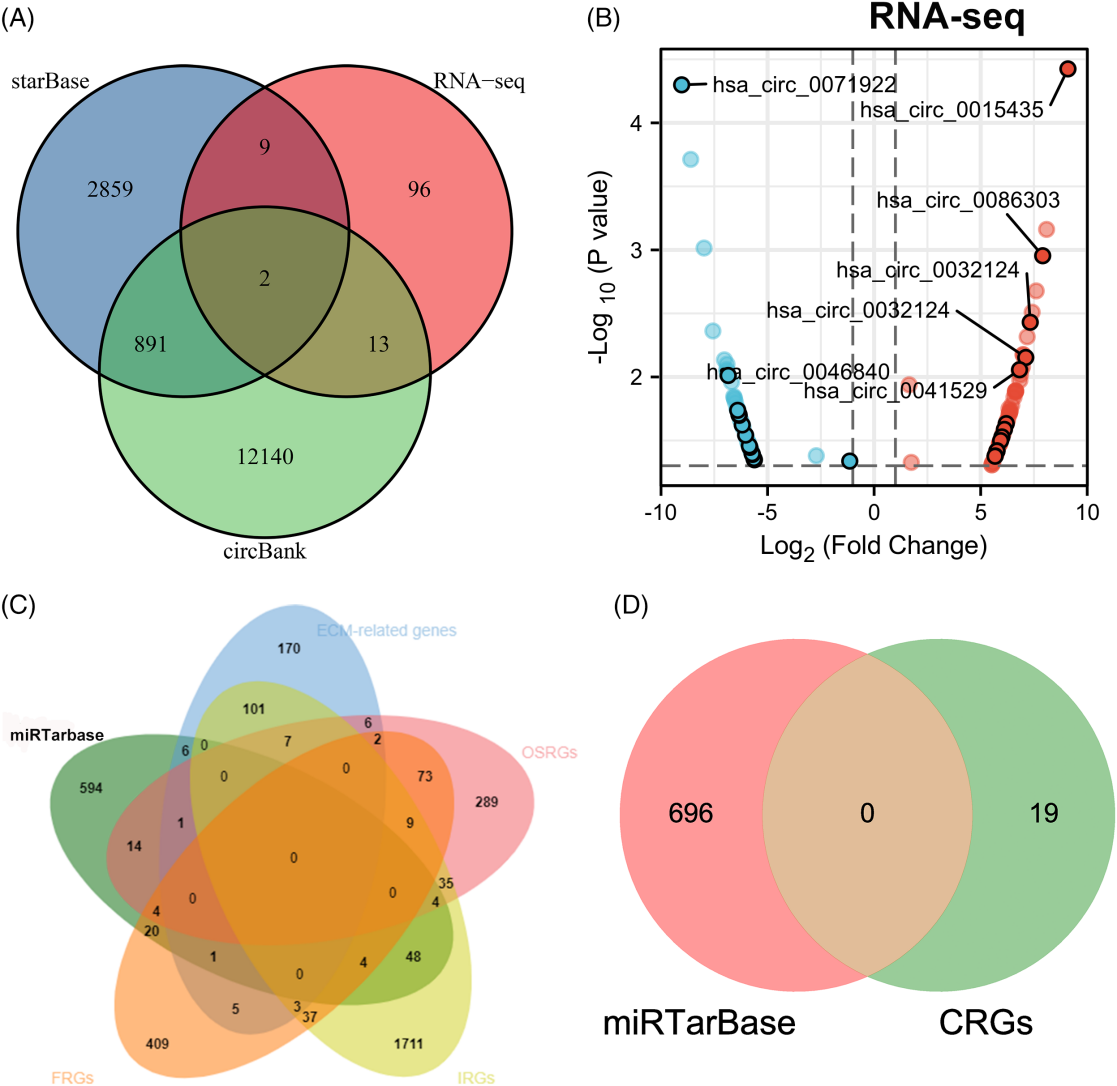
Identification of the upstream circRNAs and downstream mRNAs of miR‐15a‐5p. (A) Identification of the upstream IVDD‐related circRNAs of miR‐15a‐5p using Venn diagram. (B) Volcano plot shown the the differential expression of IVDD‐related circRNAs predicted in (A). (C) Identification of the downstream mRNAs of miR‐15a‐5p related to ferroptosis, oxidative stress, ECM metabolism and immune response by merging different terms. (D) Identification of the downstream mRNAs of miR‐15a‐5p related to cuproptosis.

StarBase database identifies more than 9000 miRNA‐circRNA regulatory relationships via analyzing miRNAs and circRNAs binding sites.^38^ Using the circBank^37^ and starBase^38^ databases, our study predicted the circRNA‐miRNA regulatory combinations. Subsequently, we predicted the upstream circRNAs of miR‐15a‐5p via intersecting the RNA‐seq as well as circBank and starBase data. A total of 11 overlapping circRNAs were observed between RNA‐seq and starBase data; and 15 overlapping circRNAs were observed between RNA‐seq and circBank data; among which hsa_circ_0015381 and hsa_circ_0086587 were present in three sets of data (Figure 7A). Among them, 11 DECs were upregulated and 13 DECs were downregulated in IVDD (Figure 7B).

miRTarBase is a large‐scale biological database that predominantly provides miRNA‐target interactions confirmed by biological experiments, which has undergone five revisions and enhancements.^39^ To further find the downstream mRNAs of miR‐15a‐5p that related to the IRGs, FRGs, CRGs, OSRGs, and ECM‐related genes, we merged different terms. Through the Venn analysis, we identified 101 mRNAs in total, of which no mRNAs related to cuproptosis was found (Figure 7C,D).

### 3.7 Construction of circRNA‐miRNA‐mRNA regulatory network in IVDD

**FIGURE 8:**
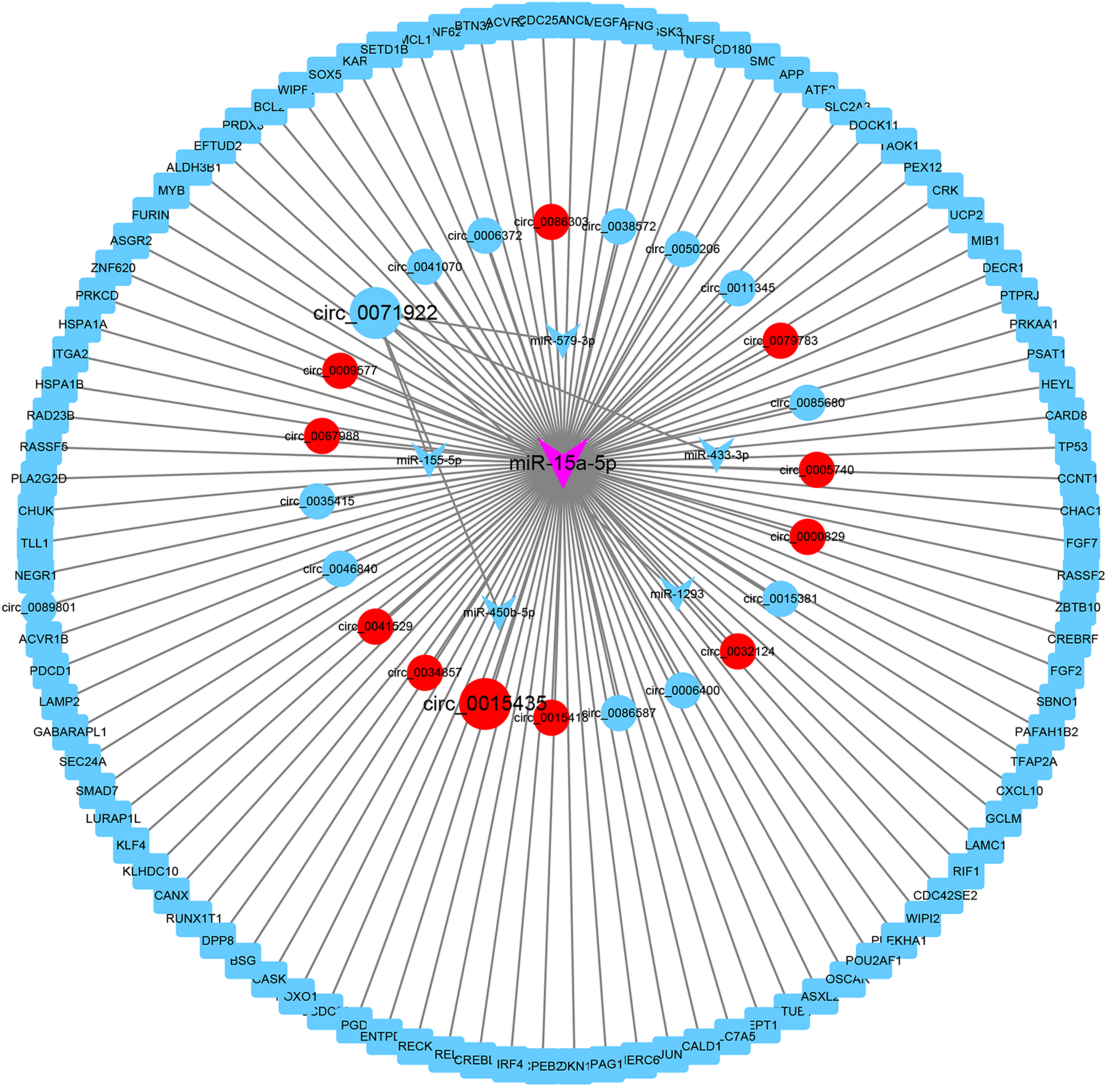
Construction of circRNA‐miRNA‐mRNA regulatory network in IVDD. The ceRNA network encompassing 24 circRNAs, 6 miRNAs, and 101 mRNAs, which was constructed by Cytoscape software.

To further elucidate the interactions between circRNAs, miRNA, and mRNAs, a circRNA‐miRNA‐mRNA ceRNA regulatory network was constructed by combining data from circRNAs and mRNAs with miRNA data. First, circ_0071922 was the top downregulated DECs in IVDD (Figure 1E). Second, we predicted hsa‐miR‐15a‐5p, hsa‐miR‐1293, hsa‐miR‐579‐3p, hsa‐miR‐433‐3p, hsa‐miR‐155‐5p, and hsa‐miR‐450b‐5p were the downstream miRNAs of circ_0071922, of which miR‐15a‐5p was the top upregulated miRNA in IVDD (Figure 6D). Third, 24 DECs was predicted as the upstream circRNAs of hsa‐miR‐15a‐5p, and 101 key mRNAs was predicted as the downstream mRNAs of hsa‐miR‐15a‐5p (Figure 7A–C). Taken together, the complete ceRNA network was constructed by Cytoscape software based on the above findings (Figure 8).

### 3.8 Overexpression of circ_0071922 can alleviate IVDD in a rat model

The position of the tail is shallow, with high mobility, and is easy to touch outside the body.^26^ Furthermore, our previous article have developed a manual palpation method to pinpoint the intervertebral disc levels of rats tail without using x‐ray examination.^26^ Increasing evidence have demonstrated that the tail puncture is the most commonly used model for constructing IVDD compared with lumbar disc puncture model.^26^, ^46^, ^47^ Thus, we intend to construct IVDD model via tail puncture method.

**FIGURE 9:**
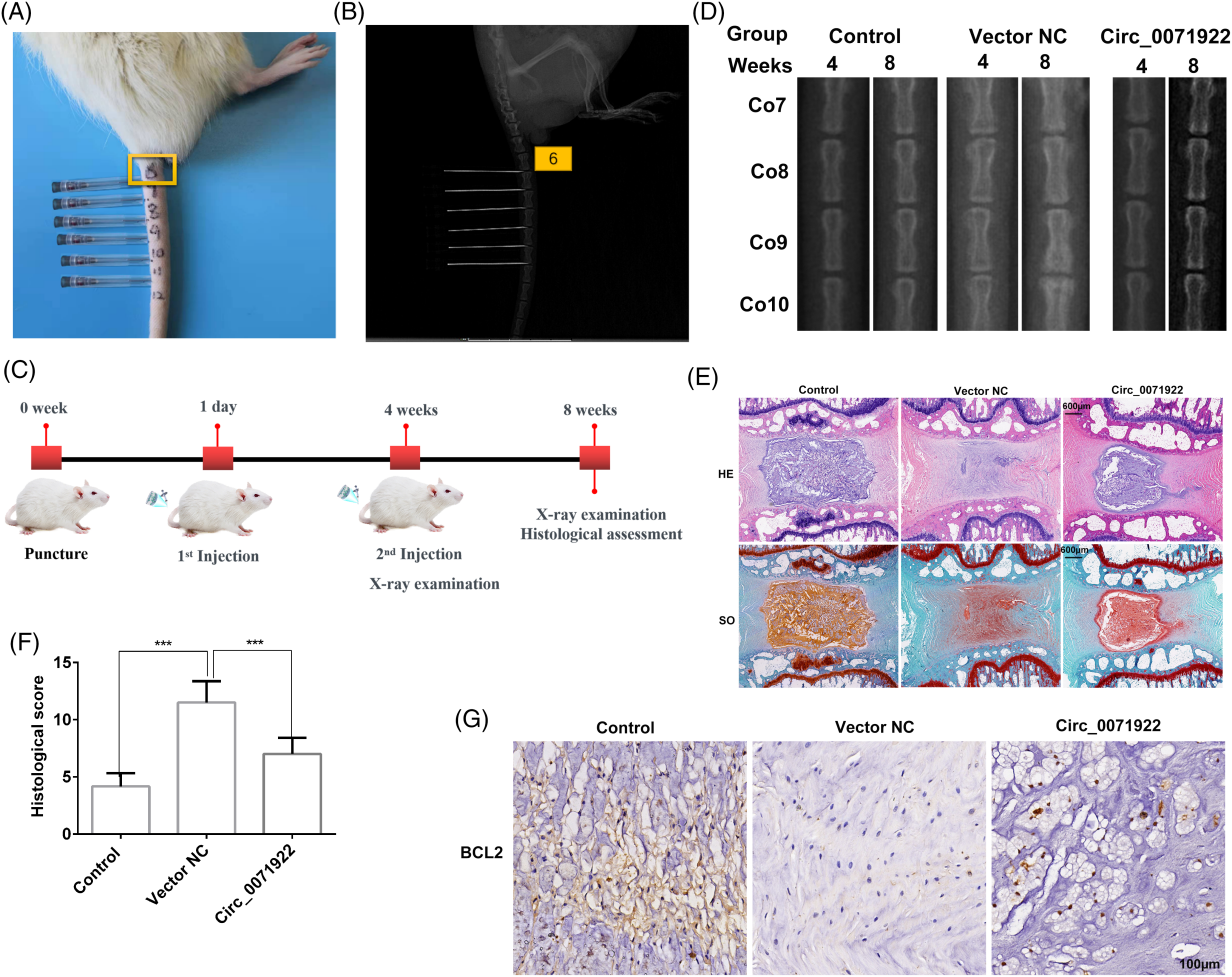
Overexpression of circ_0071922 can alleviate IVDD in a rat model. (A) Locating the intervertebral discs of rats' tail by needle puncture. (B) X‐ray shown the needle puncture to the intervertebral discs. (C) The treatment and detection experimental schedule. (D) The degree of IDD was evaluated using x‐ray examination at 4 and 8 weeks after surgery. (E) The representative histopathological images of intervertebral discs with HE and safranin O/fast green staining at 8 weeks in the control, Vector NC and circ_0071922 experiments groups. Scale bar = 600 μm. (F) Histological scoring results of HE and safranin O/fast green staining. (G) Immunohistochemical staining detects the expression level of BCL2 at 8 weeks in the control, Vector NC and circ_0071922 experiments groups. Scale bar = 100 μm.

To confirm the roles of circ_0071922 in IVDD, we conducted the animal experiments. Our previous study found that a 31G needle can be used to construct rat IVDD model.^26^ In addition, Ji and colleague also demonstrated that this needle does not cause acute inflammation response.^46^ Thus, we used a 31G needle to puncture the Co7/8 and Co9/10 intervertebral discs of the rat tail to construct a model of IVDD using two‐hand palpation method, with the Co8/9 as control (Figure 9A,B). X‐ray examination confirmed that the punctured intervertebral discs height were reduced until 4 weeks after the puncture, indicating that the model was successfully constructed (Figure 9D). Adenoviral containing circ_0071922 overexpression vector were injected into the punctured intervertebral discs at 1 day and 4 weeks after surgery (Figure 9C). The x‐ray examination results after 8 weeks of injection confirmed that the height of intervened intervertebral discs were recovered in the circ_0071922 treatment group (Figure 9D). Hematoxylin & Eosin (H&E) and Safranin O‐fast green staining showed clear boundaries between nucleus pulposus and annulus fibrosus, and the large nucleus pulposus cells population were located in the central area of the oval nucleus pulposus, which contained a multitude of proteoglycan‐rich matrix in the control group, whereas the negative control group exhibited the decreased nucleus pulposus volume and cells density together with vanishing nucleus pulposus‐annulus fibrosus boundary (Figure 9E,F). Notably, we also discovered that the circ_0071922 treatment group had a significant recovery of nucleus pulposus volume (Figure 9E,F). Furthermore, B‐cell lymphoma 2 (BCL2) is an anti‐apoptotic protein, which is not only an oxidative stress related gene but also the target gene of miR‐15a‐5p (Figures 7C and 8). Thus, we detect the expression level of BCL2 at 8 weeks after surgery in the control, Vector NC and circ_0071922 experiments groups rats using immunohistochemical staining. The result unveiled that overexpression of circ_0071922 can promote the expression level of BCL2 in rats NP tissues (Figure 9G). Thus, these data revealed a therapeutic role for circ_0071922 in alleviating IVDD progression.

## 4 DISCUSSION

A multitude of DECs have been already identified in human IVDD diseases, and their biological functions and molecular regulatory mechanisms have been gradually clarified with the development of second generation RNA sequencing and bioinformatics technology. As a major type of non‐coding regulatory RNAs, compelling evidence has unveiled that circRNAs powerfully initiated or affected the pathological processes of IVDD, including nucleus pulposus cell apoptosis, pyroptosis, senescence, and growth as well as inflammatory response and ECM metabolism.^22^, ^23^, ^24^, ^25^, ^29^, ^47^ Our previous work discovered that circ‐FAM169A, circ_0004354, and circ_0040039 are involved in the regulation of IVDD through mediating different pathological processes in a miRNA‐dependent manner.^22^, ^23^, ^24^ Wang et al.^47^ demonstrated that circKIF18A can protect nucleus pulposus cell from oxidative stress‐induced cells cycle arrest, senescence and ECM degradation. Moreover, circRNAs have highly stability and conservation due to its unique covalent closed‐loop structure.^48^ Based on these characteristics of circRNAs, accumulating number of translational investigations suggested that the DECs can be used as a new biomarker to diagnose IVDD and predict its clinical results.^24^, ^29^, ^47^ Given that cells death (such as cuproptosis and ferroptosis), oxidative stress, ECM metabolism, inflammatory and immune response were the key pathomechanism of IVDD.^8^, ^12^, ^14^, ^19^, ^20^ However, no study was made to demonstrate the DECs‐mediated cuproptosis, ferroptosis, oxidative stress, and immune response in IVDD. Thus, more study is urgently needed to decode the mysteries of IVDD by elucidating the novel functions and mechanisms of DECs in IVDD.

To find circRNAs that related to cuproptosis, ferroptosis, oxidative stress, ECM metabolism, and immune response, we conducted second generation RNA sequencing. A total of 432 DECs were identified in IVDD, including 264 upregulated and 168 downregulated DECs. On the one hand, we analyzed the host genes of DECs and found 11 genes (PRKCA, COL1A1, PSEN1, COL6A2, ADAM9, P3H2, LTBP2, FN1, LTBP1, HIF1A, and SDC2) were related to ECM metabolism. In addition, ATG7 and HIF1A linked to ferroptosis and oxidative stress; FN1 and AP3B1 linked to immune response, and COL1A1 associated with immune response and oxidative stress. However, no host genes of DECs was predicted to participate in the regulation of cuproptosis. On the other hand, in consideration of circ_0071922 was the top downregulated DECs in IVDD, we predicted that hsa‐miR‐15a‐5p was the key downstream of circ_0071922. Bioinformatics analysis further observed that 24 DECs was the upstream of hsa‐miR‐15a‐5p as well as 8 ECM‐related genes (APP, FGF2, ITGA2, TLL1, LAMC1, FURIN, CASK, BSG), 23 OSRGs (MYB, BCL2, HSPA1A, JUN, MCL1, APP, UCP2, CHUK, TP53, GCLM, PRDX3, FOXO1, KLF4, PLEKHA1, HSPA1B, PRKCD, CPEB2, ALDH3B1, ZNF622, CRK, PRKAA1, LANCL1, PSMB5), 56 IRGs (ASXL2, CARD8, CREBL2, HERC6, HSPA1A, WIPF1, UCP2, VEGFA, FGF7, IFNG, RECK, PLA2G2D, ACVR1B, CALD1, PSMC4, SOX5, CANX, HSPA1B, RAD23B, CHAC1, ENTPD1, PTPRJ, RASSF5, TNFSF9, RASSF2, CREBRF, IRF4, KLHDC10, PAFAH1B2, TAOK1, CDC42SE2, ALDH3B1, EFTUD2, DOCK11, SEC24A, REL, HEYL, PRKAR2A, CD180, ASGR2, ZNF620, SBNO1, PDCD1, ZBTB10, RUNX1T1, PAG1, OSCAR, BTN3A3, CCNT1, CCDC80, ACVR2A, RIF1, NEGR1, POU2AF1, DPP8, CXCL100), 29 FRGs (ASXL2, CARD8, CREBL2, HERC6, HSPA1A, WIPF1, UCP2, VEGFA, FGF7, IFNG, RECK, PLA2G2D, ACVR1B, CALD1, PSMC4, SOX5, CANX, HSPA1B, RAD23B, CHAC1, ENTPD1, PTPRJ, RASSF5, TNFSF9, RASSF2, CREBRF, IRF4, KLHDC10, PAFAH1B2, TAOK1, CDC42SE2, ALDH3B1, EFTUD2, DOCK11, SEC24A, REL, HEYL, PRKAR2A, CD180, ASGR2, ZNF620, SBNO1, PDCD1, ZBTB10, RUNX1T1, PAG1, OSCAR, BTN3A3, CCNT1, CCDC80, ACVR2A, RIF1, NEGR1, POU2AF1, DPP8, CXCL10), and 0 CRGs were the downstream mRNAs of hsa‐miR‐15a‐5p. According to the above findings, we found 24 DECs, hsa‐miR‐15a‐5p, and 101 mRNAs might be involved in modulating ferroptosis, oxidative stress, ECM metabolism, and immune response. Furthermore, our study demonstrated that overexpression of circ_0071922 can protect against IVDD in a rat model.

A single circRNA can guide the entire cell pathway by interacting with multiple miRNAs and many target genes, a single miRNA can be regulated by multiple circRNAs and a single mRNA can also be modulated by multiple circRNAs and miRNAs, thus forming a complex and powerful circRNA‐miRNA‐mRNA regulatory network.^21^, ^48^ Therefore, circRNA‐miRNA‐mRNA based intervention seems to be the most effective therapeutic tool. Increasing studies have uncovered that the dysregulation of the circRNA‐miRNA‐mRNA interaction network has been implicated in the pathomechanism of many musculoskeletal degenerative diseases, including IVDD, osteoarthritis, rheumatoid arthritis, and ankylosing spondylitis.^48^, ^49^ Over the past few years, the investigations of circRNA‐miRNA‐mRNA network has become one of the most significant mechanisms study of IVDD.^25^, ^29^, ^48^, ^49^ Functionally, growing evidences have suggested the involvement of circRNA‐miRNA‐mRNA network in IVDD by their extensive crosstalk with nucleus pulposus cell functions‐related signaling pathways.^25^, ^29^, ^49^, ^50^ Nevertheless, the study on the interaction mechanism among circRNAs, miRNAs, and mRNAs is not comprehensive in IVDD. To clearly show the interactions among circRNA, miRNA, mRNA in IVDD, we constructed circRNA‐miRNA‐mRNA regulatory networks using Cytoscape software. We can intuitively see many circRNA‐miRNA‐mRNA networks interactions might mediate the functional changes of nucleus pulposus cells by regulating the IRGs, FRGs, OSRGs and ECM‐related genes. However, further studies need to be conducted to demonstrate whether these dysregulated circRNA‐miRNA‐mRNA networks really modulate the nucleus pulposus cells ferroptosis, oxidative stress, ECM metabolism, and immune response.

However, there remained shortcomings in this study. The clinical sample size used for RNA‐sequencing was relatively small, and we expect a larger sample size to expand and improve our conclusions. Thus, more normal and degenerative nucleus pulposus tissues need to be collected to verify the true expression levels of circ_0071922 as well as hsa‐miR‐15a‐5p and its key target mRNAs in IVDD. In addition, we only sequenced and analyzed circRNAs and miRNAs expression profiles, but did not sequenced the expression of mRNAs in IVDD. The lack of molecular biology is another disadvantage of this study, so further investigations are needed to demonstrate the functions and mutual regulatory mechanisms of the circRNA‐miRNA‐mRNA networks in IVDD in the future.

## 5 CONCLUSION

In conclusion, this study identified 264 upregulated and 168 downregulated DECs using RNA‐sequencing, of which circ_0015435 was most obviously upregulated and circ_0071922 was most obviously downregulated in IVDD. Then we found that hsa‐miR‐15a‐5p was the key downstream of circ_0071922, and hsa‐miR‐15a‐5p was the top upregulated miRNA in IVDD. We also predicted 56 IRGs, 29 FRGs, 23 OSRGs, and 8 ECM‐related genes are the targets mRNAs of hsa‐miR‐15a‐5p by bioinformatics analysis. Through integrating analyzed data, we successfully constructed a ceRNA network encompassing 24 circRNAs, 6 miRNAs, and 101 mRNAs. Additionally, we also demonstrated that overexpression of circ_0071922 can alleviate IVDD progression in a rat model. The findings of this study suggested that circ_0071922‐miR‐15a‐5p‐mRNA signaling network might affect IVDD by modulating the nucleus pulposus cells ferroptosis, oxidative stress, ECM metabolism, and immune response. These data are expected to provide potential biomarkers and effective therapeutic targets for IVDD management.

## AUTHOR CONTRIBUTIONS

Yongjin Li, Xiaolong Chen, and Shibao Lu contributed to the concept and the design of the article. Yongjin Li wrote the manuscript. Wenzhi Sun and Baobao Wang drew the tables and figures. Yongjin Li, Junzhe Ding, and Chao Kong helped with the bioinformatics analysis. Feng Hu collected the patients and healthy donors' clinical information and tissues. Jianhua Li conducted the animal experiments. Xiaolong Chen and Shibao Lu critically reviewed the manuscript. All authors read and approved the final manuscript.

## CONFLICT OF INTEREST STATEMENT

The authors declare that this research was conducted in the absence of any commercial or financial relationships that could have appeared to influence the work reported in this paper.

## Supporting information


**Table S1.** The lists of OSRGs, FRGs, CRGs, IRGs, and ECM related genes.

**Table S2.** The detail information of 129 DECs.

**Table S3.** The host genes of circRNAs.

## Data Availability

The data that support the findings of this study are available from the corresponding author upon reasonable request.
